# Detection of abemaciclib, an anti-breast cancer agent, using a new electrochemical DNA biosensor

**DOI:** 10.3389/fchem.2022.980162

**Published:** 2022-10-21

**Authors:** Zimeng Lei, Merim Alwan, Hassan Thoulfikar A. Alamir, Hussein Humedy Chlib Alkaaby, Sinan Subhi Farhan, Sura A. Awadh, Usama S. Altimari, Hawra’a Fadhel Abbas Al-Baghdady, Athmar Ali Kadhim, Maytham T. Qasim, Ali Hussein Adhab, Abuzar Nekuei

**Affiliations:** ^1^ School of International Education, Beijing University of Chemical Technology, Beijing, China; ^2^ Medical Lab. Techniques Department, College of Medical Technology, Al-Farahidi University, Baghdad, Iraq; ^3^ Faculty of Pharmacy Department of Pharmaceutics, University of Al-Ameed, Karbala, Iraq; ^4^ Al-Manara College for Medical Sciences, Maysan, Iraq; ^5^ The University of Mashreq, Baghdad, Iraq; ^6^ Department of Anesthesia, Al-mustaqbal University, Babylon, Iraq; ^7^ Al-Nisour University College, Baghdad, Iraq; ^8^ Hawra’a Fadhel Abbas AL-Baghdady, College of Dentistry, The Islamic University, Najaf, Iraq; ^9^ Medical Laboratories Teachniques, Hilla University College Babylon, Babylon, Iraq; ^10^ Department of Anesthesia, College of Health and Medical Technology, Al-Ayen University, Thi-Qar, Iraq; ^11^ Department of Medical Laboratory Technics, Al-Zahrawi University College, Karbala, Iraq; ^12^ Islamic Azad University of South Tehran Branch, Tehran, Iran

**Keywords:** abemaciclib, DNA biosensor, anti-breast cancer agent, voltammetry, modified electrode

## Abstract

Detection of DNA molecules and possible chemotherapy-induced changes in its structure has been the goal of researchers using rapid, sensitive and inexpensive approaches. Therefore, the aim of this study was to fabricate a new electrochemical DNA biosensor using pencil graphite electrodes modified with polypyrrole/Ce doped hexagonal nickel oxide nanodisks or PP/Ce-doped H-NiO-ND composites for determination of Abemaciclib (AMC) and ds-DNA molecules. The DNA biosensor was prepared by immobilizing ds-DNA on the surface of PP/Ce-doped H-NiO-ND/PGE. Differential pulse voltammetry (DPV) was used to electrochemically detect AMC. The results elucidate the extremely high sensitivity of the ds-DNA/PP/Ce-doped H-NiO-ND/PGE biosensor to AMC, with a narrow detection limit of 2.7 nM and a lengthy linear range of 0.01–600.0 μM. The admirable performance of as-fabricated biosensor could be related to the active reaction sites and the unique electrochemical response related to the nanocomposites by enhancing ds-DNA stabilization and accelerating electron transfer on the surface of electrode.

## Introduction

Breast cancer (BC) is the main reason for cancer-related death among women around the world, with 2.6 million new cases and about 685,000 deaths. This cancer mainly leads to the death of about 30% of cancer patients according to global mammography data in 2021, one of the four cancers diagnosed among women (Ferlay et al., 2015).

**FIGURE 1:**
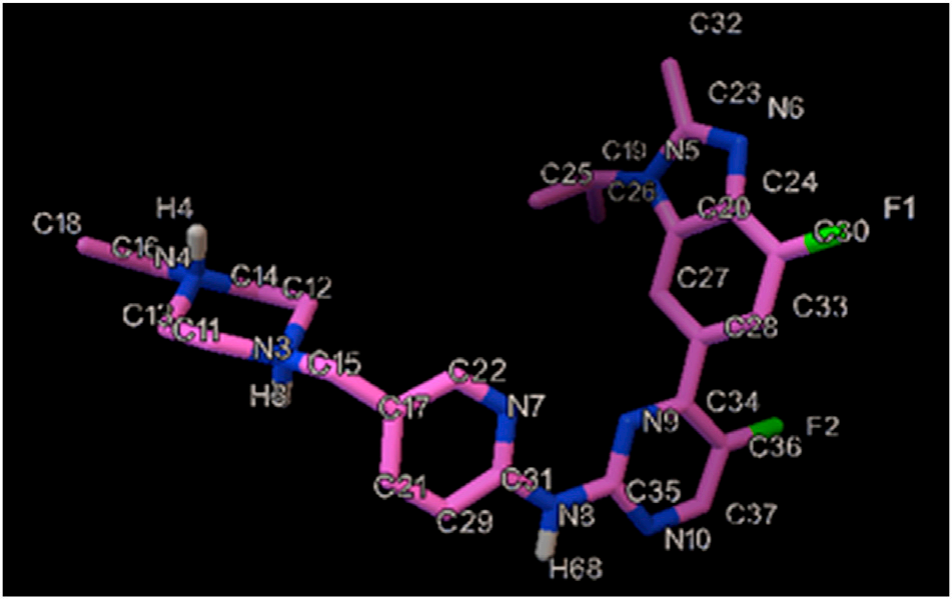
The illustration of the chemical structure of AMC with the numbered atoms.

One of the common treatments for some breast cancers has been approved to be Abemaciclib (AMC) (Figure 1), especially in combination (Margaryan et al., 2022), which is able to cross the blood-brain barrier (BBB) and inhibit tumor growth (Raub et al., 2015). Findings of a study on a physiologically oriented pharmacokinetic model recommended that AMC be used to treat brain cancer, which is preferable to other CDK4/6 inhibitors like palbociclib and ribociclib (Li et al., 2021). According to a phase II clinical trial on patients experiencing brain metastasis, the AMC therapeutic concentrations were confirmed in metastatic brain tissue, as a promising finding for the search for new combinations based on this agent. It is true that AMC is widely prescribed in chemotherapy, but evidence has documented some complications, some of which are constipation, stomach pain, loss of appetite, nausea, vomiting, hair loss, and sores on the throat, lips or mouth (Tolaney et al., 2020).

Accordingly, it is critical to adjust the dose of this drug during the chemotherapy process. Hence, there have been previously several analytical approaches for this purpose (Karimi-Maleha et al., 2022). Although all of these approaches have admirable advantages, such as rapid response time and portability, special attention has been paid to electrochemical methods in detecting anticancer agents. However, electrochemical sensors have drawbacks in quantifying drug compounds such as low reduction/oxidation current and high overpotential, which must be circumvented. The sensor functionalization can be prospective method to monitor the conventional drug dosage (Bayraktepe, 2020; Fathi et al., 2020; Foroughi et al., 2021a; Foroughi et al., 2015; Li et al., 2022; Maaref et al., 2018; Moarefdoust et al., 2021). Nanomaterials have shown specific physical and chemical characteristics in a variety of fields (Akca et al., 2021; Farvardin et al., 2020; Foroughi and Jahani, 2022; Foroughi and Ranjbar, 2017; Mei et al., 2022; Salajegheh et al., 2019; Zhou et al., 2022).

Diverse p- and n-type metal-oxide nanomaterials (MONs) have been proposed promising options for electron mediator during electrochemical sensing (Jahani et al., 2022; Zhou et al., 2018).

Transition metal oxide such as nickel oxide (NiO) has acted well as an electrocatalyst in areas such as electrochemical sensors, batteries, supercapacitors and water splitting (Goel et al., 2022; Mohandesi et al., 2022; Sethi et al., 2021; Sun et al., 2022). Exceptional performance with appreciable sensing ability can be achieved *via* NiO due to the concurrent Ni^2+^ active sites, impressive electrochemical stability and acceptable adsorption capability (Veeman and Karuppuchamy, 2022). One of the well-proved modifiers is metal oxide nanocomposite in electrochemical sensing systems (Foroughi et al., 2021b).

Lanthanides extensively explored in the forms of oxide, oxycarbonate, hydroxide, oxychloride or phosphate, with the applications in optoelectronic, solid electrolyte, phosphors, sorbent and catalytic equipment (Dakhel, 2009; Ferhi et al., 2008; Huang et al., 2000; Imanaka et al., 2002; Tokunaga et al., 1997). For instance, cerium has shown unique attributes as material for the fabrications of catalysts, optical filters and high-k gate dielectrics (Chellammal Gayathri et al., 2022; Fudala et al., 2022; Kuznetsov et al., 2022). The majority of such advanced functions are based on the structures and compositions with high sensitivity to bonding modes of rare earth ions or atoms (Mu and Wang, 2011). Accordingly, two integrated perspectives were applied in our study for development of ultra-sensitive electrochemical, including 1) ds-DNA as an element for enhancement of AMC biosensor selectivity, and 2) polypyrrole/Ce doped hexagonal nickel oxide nanodisks (PP/Ce-doped H-NiO-ND) as a composites for enhancement of AMC biosensor sensitivity.

## Materials and methods

### Chemicals and devices

Nickel nitrate hexahydrate, cerium nitrate hexahydrate, NH_4_OH and hexamethylenetetramine (HMTA) were Merck, and AMC and ds-DNA (Calf Thymus) were Sigma-Aldrich. The optimized detection of AMC was monitored by differential pulse voltammograms (DPVs) using Potentiostat/Galvanostat (Ivium-Vertex) device. The renewable PGE that was described in the study of Ensafi and co-workers (Ensaﬁ et al., 2012) were used in all experiments. A Noki pencil Model 2000 (Japan) was used as a holder for Pentel graphite leads. Electrical contact with the lead was obtained by soldering a metallic wire to the metallic part. The pencil was hold vertically with 12 mm of the lead extruded outside (10 mm of which was immersed in the solution). The pencil leads were used as received. The reference electrode was saturated calomel electrode (SCE) and the counter electrode was a platinum wire. Mira 3-XMU field emission-scanning electron microscopy (FE-SEM) with energy dispersive X-ray spectroscopy (EDS) was used for the morphological exploration of Ce-doped H-NiO-ND composite. The patterns of PANanalytical Xpert Pro X-ray diffraction (XRD) with Cu-Kα radiation of *λ* = 1.54178 Å at the range between 2 and 80° were used to characterize crystallinity and crystal phases. The AMC was homogeneously suspended in buffer solution to obtain 0.01 M AMC stock solution.

### Production of Ce-doped hexagonal nickel oxide nanodisks

Ce-doped hexagonal nickel oxide nanodisks were prepared by dissolving cerium nitrate hexahydrate (Ce(NO_3_)_3_·6H_2_O, 1.0 g, 0.0023) and nickel nitrate hexahydrate (Ni(NO_3_)_2_.6H_2_O, 2.9 g) and hexamethylenetetramine (1.4 g) in DDW (100 ml) while stirring rigorously for 15 min. The solution pH was adjusted to 9 using some drops of NH_4_OH, followed by stirring vigorously for 60 min. The solution was then sealed and heated up to 140°C for 8 h in a Teflon-lined stainless steel autoclave, followed by cooling down to room temperature. The resultant precipitate was washed with DDW/ethanol and dried at ambient temperature overnight, followed by calcination at 600°C for 2 h. Diverse techniques were applied to characterize the final product.

### Production of PP/Ce-doped H-NiO-ND/PGE

The pencil graphite electrode (PGE) was cleaned by acetate buffer (0.1 M), followed by ultra-sonicated for 2 min. Then, the surface of electrode was pre-treated in a 0.5 M HCl by cyclic voltammetry (CV) at the potential of 0–1.2 V during 20 cycles. The prepared Ce-doped H-NiO-ND composite (2 mg) was dissolved in water (10 ml) under subsequent ultra-sonication for 30 min, and subsequently appending 0.1 M pyrrole. The CV at the potential of 0–0.8 V with the scan rate of 100 mV/s during 30 cycles was applied for the pyrrole electro-polymerization on the surface of PGE.

### ds-DNA immobilization on PP/Ce-doped H-NiO-ND/PGE surface and DNA detection

The ds-DNA immobilization was carried out by passive adsorption. Thus, the PP/Ce-doped H-NiO-ND/PGE was added to ds-DNA solution (30.0 mg/ml) in acetate buffer (0.5 M, pH 4.8) for diverse periods with the potential of +0.50 V. The electrodes were all rinsed thoroughly *via* ABS for removal of non-attached DNA molecules to obtain ds-DNA/PP/Ce-doped H-NiO-ND/PGE, followed by being air-dried.

### Interaction of AMC with ds-DNA/PP/Ce-doped H-NiO-ND/PGE, and process of sensing

The modified ds-DNA/PP/Ce-doped H-NiO-ND/PGEs and variable levels of AMC were appended to TBS (10.0 mM) at the pH 7.2, followed by incubating for 30 min, rinsing *via* buffer solution and drying. The binding of AMC with DNA on ds-DNA/PP/Ce-doped H-NiO-ND/PGE was determined by a voltammetric transduction in AMC-free ABS at the pH 4.8 based on the DPV. Then, the guanine oxidation peak currents as analytical marker exhibited a decrease with washing the concentrations of AMC. This trend was done in the same way by a novel ds-DNA biosensor. More sensitive electrochemical signals were investigated under diverse experimental conditions such as the influence of ds-DNA + AMC concentration, and ds-DNA stabilization time and interaction time on sensor performance.

### Analysis of biological and pharmaceutical specimens

The real specimens were AMC injection (1 mg/ml) collected from a domestic pharmacy in China, which were analyzed. Initially, dilution of 1 ml AMC injection has been done to 10 ml with ABS pH of 4.8. Afterward, distinct volumes of the diluted solutions were poured in a volumetric flask (25 ml). Finally, the supposed technique has been employed for analyzing AMC contents through the standard addition approach.

AMC is released to the blood serum (or urine) of healthy volunteers from a medical laboratory in China. Then, the recovery of the drug was achieved using the suggested approach. In the blood serum (or urine) samples (5.0 ml), 5 ml of methanol was released for removing proteins. The precipitated proteins were separated by 3 min centrifugation at 5,000 rpm after 2 min sample vortexing. A milli-pore filter (0.45 µm) was used to filter the clear supernatant layer. Upon the transfer of a 2.5 ml volume of serum (or urine) into a vial consisting of 22.5 ml AMC with a pH of 4.8, a specific amount of stock solution of AMC were poured into the vial, then transported the mixture into an electrochemical cell and obtained ΔE was recorded. Standard addition method has been run for determining contents of AMC samples. After that, contents of AMC were specified utilizing calibration curve.

In addition, to measure AMC in the real sample, fresh milk has been acquired from a local market. Firstly, a certain amount of hydrochloric acid (0.01 M) has been added to 5.0 ml liquid milk and stirred for 10 min. Then, the prepared solution has been centrifuged for 20 min at 8,000 rpm to obtain the supernatant. The *t*-test was elaborated to compare the precision of method.

### Electrochemical approach

Cyclic voltammetry (CV) and differential pulse voltammetry (DPV) was applied for electrochemical studies and quantification of AMC.

CV is performed to a KCl (0.1 M) the presence of [Fe(CN)_6_]^3−/4−^ (0.35 mM), starting at the equilibrium potential in anodic direction using a potential window of ^−^0.1 to 0.55 V at different scan rates. Anodic peaks are analyzed in order to stablish the relation between the maximum current intensity of the anodic peaks with the scan rate.

In order to achieve the higher analytical response (anodic current), the optimal conditions for DPV measurements were as follow: ABS, pH 4.8, scan rate of 50 mV/s, modulation amplitude of 0.02505 V, modulation time of 30 ms, interval time of 200 ms, step potential of 10 mV, initial potential = 700 mV and end potential of 1,000 mV. To achieve the DP voltammograms of AMC, appropriate volumes of the stock solutions of drugs were added to the cell containing supporting electrolyte on total bulk of 25 ml.

### Molecular docking study

A molecular docking investigation was conducted as part of a biological assay to predict the mode of binding of AMC anticancer drug inside the DNA receptor. For this goal, the intercalation mode was proposed and simulated, in which DNA structures with hexamer d (CGATCG)2 sequences featuring 1Z3F, was employed.

For giving the most stable geometric of Abemaciclib, the structure optimizing calculation was performed by Gaussian 09 at the 6–31 G** level by using the B3LYP hybrid density functional theory (DFT). Autodock4.2.6 was applied by a semi-flexible docking technique. In this study, all Abemaciclib bonds were set free while DNA kept rigid. The grid point spacing of 0.375 Å and the grid map with 80 Å *×* 80 Å *×* 80 Å points were created. The docking included the maximum 25,000,000 energy calculations, and 200 separate docking runs were performed utilizing the Lamarckian genetic algorithm local search technique (Niroomand et al., 2017; Yinhua et al., 2020).

## Results and discussion

### Analysis of Ce-doped H-NiO-ND characteristics

As-fabricated Ce-doped H-NiO-ND was examined for crystallinity, phase and morphology as follows. The XRD patterns (Supplementary Figure S1) regarding crystal structure verified an acceptable crystallinity. The peaks at 36.93°, 43.12°, 62.73°, 75.61° and 79.38° were related to the planes of (111), (200), (220), (311), and (222), respectively. The XRD data are evidence of cubic form of NiO (JCPDS No: 78-0643) (Zhou et al., 2018). Greater shift in degree is evident in the (111), (200) and (220) peaks for Ce-doped H-NiO-ND probably because of Ce incorporation in NiO lattice, leading to internal strain caused by bigger atomic radii (185 p.m.) when comparing with Ni (124 p.m.). There was no secondary peak in XRD corresponding to dopant, meaning the production of single phase NiO nanostructures. According to XRD findings, the diffraction peaks corresponded to NiO only. Based on the XRD results, the Scherer equation of D = Kλ/ßcosθ (Jahani, 2018; Xu et al., 2015) was used to calculate the Ce-doped H-NiO-ND crystallite size, in which *λ* stands for the used X-ray wavelength (1.541 Å), β for the peak width at half maximum (FWHM) and θ for the Bragg diffraction angle, which was 94 nm.

The morphological analysis of Ce-doped H-NiO-ND was done by FE-SEM, the results of which are presented in Supplementary Figures S2A,B. Based on the micrographs, well-defined hexagonal nanodisks are evident, in large-quantity. Each nanodisk is linked to its adjacent nanodisk through one of its surfaces because of its very high growth density. The nano-disks had smooth, clean surfaces with sharp edges. The nanodisk size is not uniform, so some nanodisks are completely hexagonal in shape, and some have a deformed hexagonal shape. The mean diagonal of nanodisks ranged from 230 to 400 nm, although the micrograph shows some larger nanodisks. The mean typical nanodisk thickness was 30 ± 10 nm, as seen in Supplementary Figure S2B.

Supplementary Figure S3 illustrates Ce-doped H-NiO-ND EDS analysis. According to the EDS analysis, the compositions contained only Ni, Ce and O, with no impurity.

### Analysis of modified electrode characteristics

Great attention has been recently attracted towards disposable PGEs in DNA and DNA-antitumor drug interplay due to impressive merits like practicability, reproducibility high availability, adaptability low cost and simple modification (Dogan-Topal and Ozkan, 2011a; Tig et al., 2016).

**FIGURE 2:**
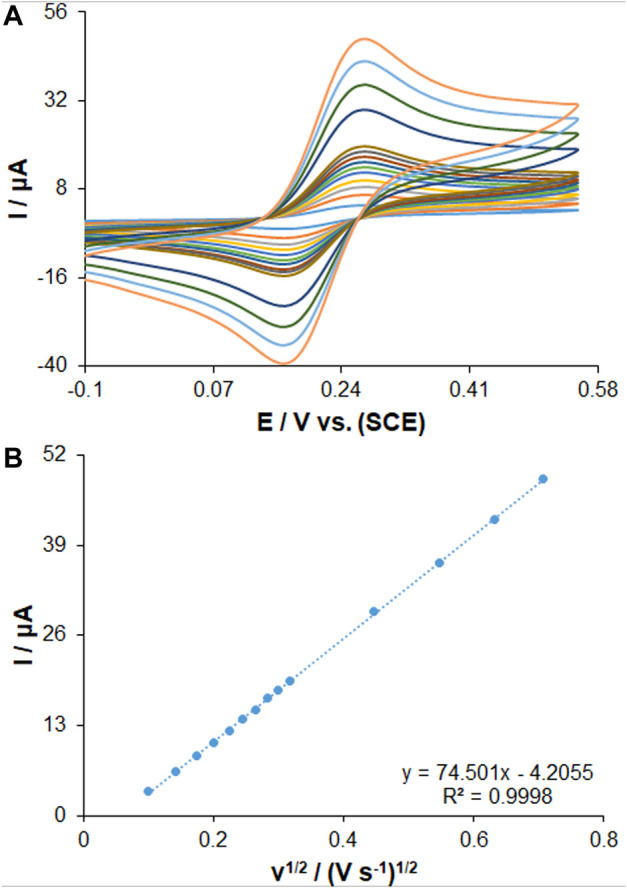
**(A)** CVs of PP/Ce-doped H-NiO-ND/PGE in the presence of 0.35 mM [Fe(CN)_6_]^3−^ solution in aqueous 0.1 M KCl at various scan rates (from inner to outer curve): 10, 20, 30, 40, 50, 60, 70, 80, 90, 100, 200, 300, 400 and 500 mV/s. **(B)** The plot of peak currents vs. υ^1/2^.

Three electrodes consisting of bare PGE, PP/H-NiO-ND/PEG and PP/Ce-doped H-NiO-ND/PEG were investigated using cyclic voltammetry (CV) in a 0.1 M solution of KCl including a redox probe [0.5 mM K_3_Fe(CN)_6_ solution and 0.35 mM K_4_Fe(CN)_6_] for investigating the efficiency of these electrodes. The electroactive surface areas of bare PGE, PP/H-NiO-ND/PEG and PP/Ce-doped H-NiO-ND/PEG (Figure 2) were determined using Randles-Sevcik equation (Eq. 1) (Bard and Faulkner, 2001).

$$I_{p}=\pm \left(2.69\times 10^{5}\right)n^{3/2}AD^{1/2}Cv^{1/2}$$ (1)

Where D shows the diffusion coefficient (cm^2^s^−1^), A stands for the electrode surface area (cm^2^), C stands for the [Fe(CN)_6_]^3−/4−^ (0.35 mM) concentration, n shows the number of electrons consisted in the process (*n* = 1), Ip stands for the intensity of peak current (A), and ν shows the rate of scan (V s^−1^). The electroactive surface area amounts for the bare PGE, PP/H-NiO-ND/PEG and PP/Ce-doped H-NiO-ND/PEG were predicted 0.082, 0.22 and 0.29 cm^2^, respectively. The obtained outcomes demonstrated that the PP/Ce-doped H-NiO-ND/PEG can provide a larger electroactive area and is highly suggested because it is a promising material, that can promote the sensitivity and electron transfer ability of the electrodes.

**FIGURE 3:**
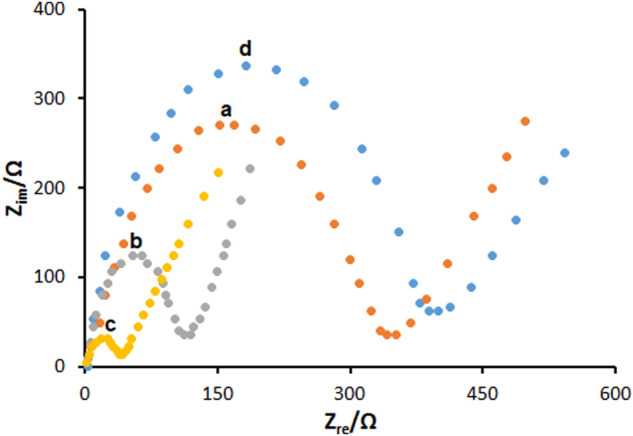
The Nyquist diagrams of impedance recorded on (a) bare PGE, (b) PGE coated with PP/H-NiO-ND and (c) PGE coated with PP/Ce-doped H-NiO-ND, (d) 200.0 μg ml^−1^ dsDNA immobilized PP/Ce-doped H-NiO-ND/PGE in 0.1 M KCl solution containing 5.0 mM Fe(CN)_6_^3-/4-^.

The EIS has been shown to be a valid approach to determine the impedance features of the electrode-solution interface *via* the redox probe of Fe(CN)_6_^3-/4-^. The diameter of a semicircle is described by a charge transfer resistance (R_ct_) in a Nyquist diagram. These diagrams of EIS patterns on PGE during assembling surface layer are shown in Figure 3. The R_ct_ value for the unmodified PGE was obtained to be 387 ± 29 Ω, as seen in curve a, which was decreased by 149 ± 8 Ω for PP/H-NiO-ND/PGE (curve b) and 51 ± 3 Ω for PP/Ce-doped H-NiO-ND/PGE (curve c) indicating impressive electrical conductivity capable of transferring redox ions to the surface of electrode. A gradual increase was found for the R_ct_ value (408 ± 38 Ω) following the ds-DNA immobilization on the surface of PP/Ce-doped H-NiO-ND/PGE, see curve c. Therefore, there has been a decrease in the capacity of electron transfer on the surface of electrode due to non-conductive behavior of ds-DNA, thereby keeping ferro/ferricyanide ions away from reaching the electrode.

### Electrochemical performance of immobilized ds-DNA on PP/Ce-doped H-NiO-ND/PGE surface

**FIGURE 4:**
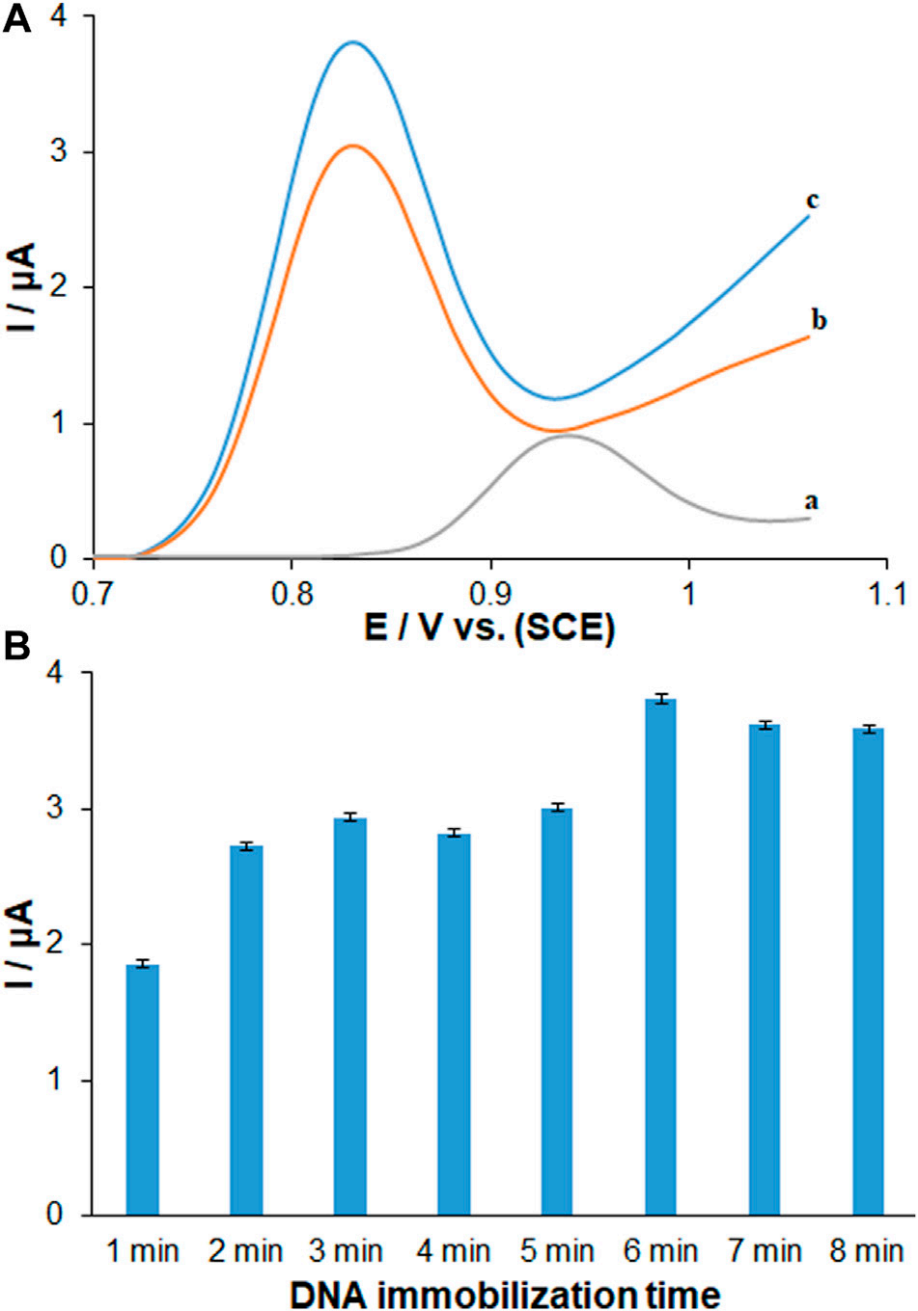
**(A)** DPVs recorded (vs. SCE) for 96.0 μg ml^−1^ dsDNA immobilized 6 min on **(a)** unmodified PGE, **(b)** PP/H-NiO-ND modified PGE and **(c)** PP/Ce-doped H-NiO-ND modified PGE in ABS pH 4.8. **(B)** Histograms corresponding to the average guanine signals after different immobilization times ranging from 1 to 8 min (*n* = 3) for dsDNA.

The ds-DNA modification on electrodes is a key stage in the production of DNA biosensor, which has a link with the process of direct adsorption. The direct electrochemistry of DNA and the comparison of DNA DPV scans between unmodified PGE and modified PP/H-NiO-ND, Ce-doped H-NiO-NDs are shown in Figure 4A. One of the markers for direct DNA detection can be electrochemical signals of electrodes to the oxidation of guanine. We calculated the peak potentials (*versus* SCE) of guanine (+0.831 V) oxidation at modified electrode (Dogan-Topal and Ozkan, 2011a). The peak currents of oxidation were elevated due to the PP/Ce-doped H-NiO-ND modification than those due to unmodified PGE, approximately 4.2 times more because of elevated composite conductivity. Higher sensitivity can be achieved following more DNA bound on film surface because of larger surface area of Ce-doped H-NiO-NDs. Hence, the binding/interfering properties of Ce-doped H-NiO-ND to biomolecules can offer commendable performance to generate a DNA-based biosensor, which means the importance of characterization.

**FIGURE 5:**
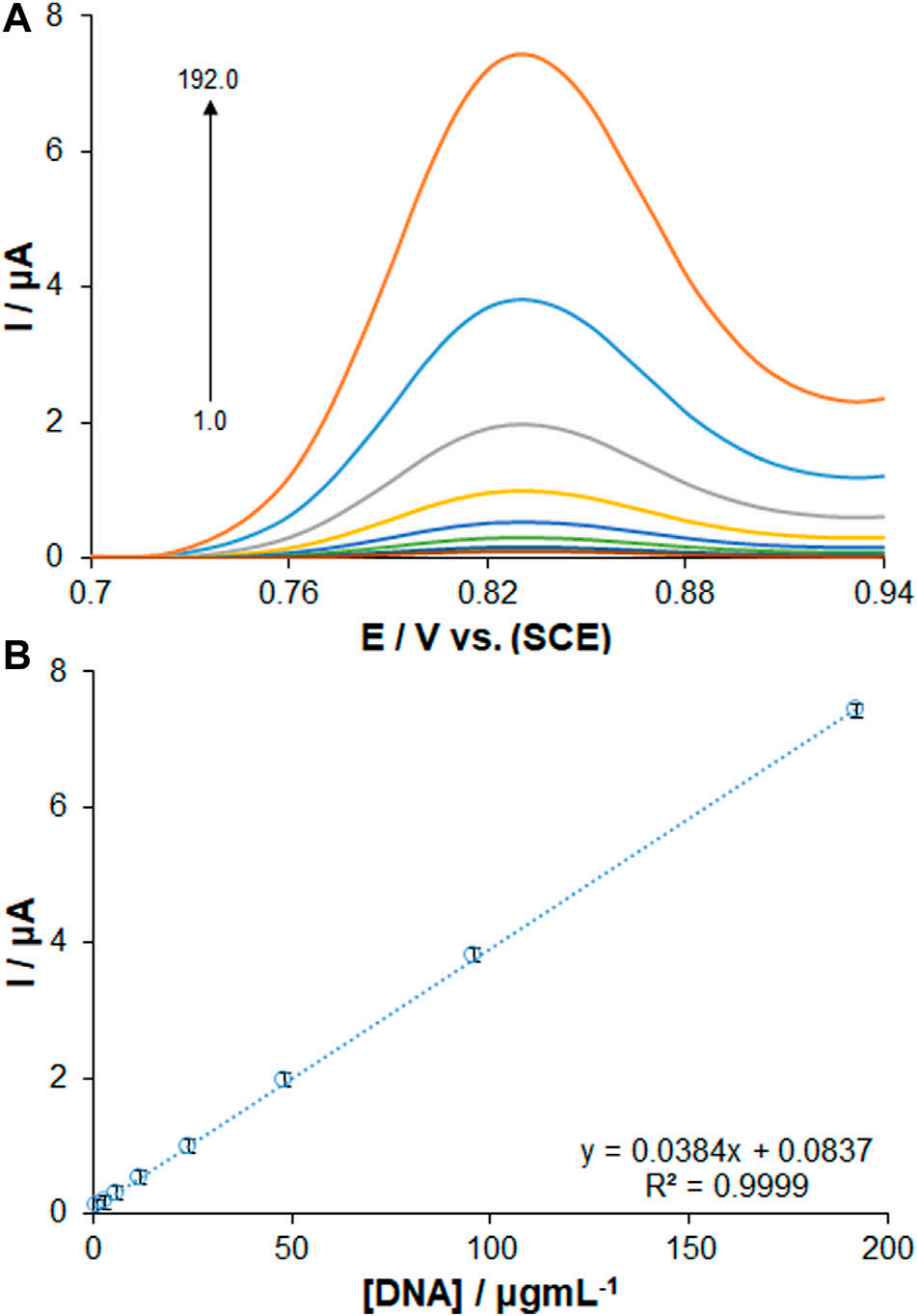
**(A)** DPVs of PP/Ce-doped H-NiO-ND/PGE for changing concentrations of dsDNA. **(B)** Calibration graph presenting the guanine oxidation signals from 1.0 to 192.0 μg ml^−1^ dsDNA in ABS pH 4.8.

The optimal sensor-DNA binding was achieved by calculating the best values of factors such as ds-DNA accumulation time and concentration. The experiments at variable DNA immobilization time from 1 to 8 min are displayed in Figure 4B. The highest peak current of guanine oxidation was seen in the accumulation time of 6 min, and no significant impact was found after long term adsorption times on the behavior owing to surface saturation, which offers the minute 6 as the optimized DNA immobilization time. The voltammetric determination of DNA according to variable concentrations is observed in Figure 5A. Figure 5B shows an excellent linearity of peak currents of guanine oxidation for the DNA immobilization on the surface of PP/Ce-doped H-NiO-ND/PGE at variable concentrations from 1.0 to 192.0 μg/ml. The limit of quantification (LOQ, 10 s/m) was 0.39 μg/ml and the limit of detection (LOD, 3 s/m) was 0.12 μg/ml *versus* the standard curve; where *s* means standard deviation of blank solution and *m* means slope of related standard curve. These data have been previously confirmed by others for DNA or DNA-drug biosensors (Erdem and Congur, 2018). The optimized DNA concentration was obtained 192.0 μg/ml. The extraordinary reproducibility was obtained following three successive DPV determinations of guanine oxidation peak at the ds-DNA concentration of 192.0 μg/ml at the 6-min accumulation time, confirmed by computing relative standard deviation (RSD) of 1.18%. Accordingly, the proposed PP/Ce-doped H-NiO-ND/PGE can be admirable option for electrochemically sensing the interface due to outstanding features like large active surface area, effective functional groups for impressive drug-DNA binding, easy preparation and commendable electrical conductivity applying electrochemical deposition.

### Exploration of ds-DNA-AMC interaction and electrochemical performance

Understanding drug-DNA interactions has hopefully been useful in determining the cause of various diseases, the mechanism of drugs’ action, and the development of new agents. This binding of the drug to DNA is of particular importance in electrochemical study.

**FIGURE 6:**
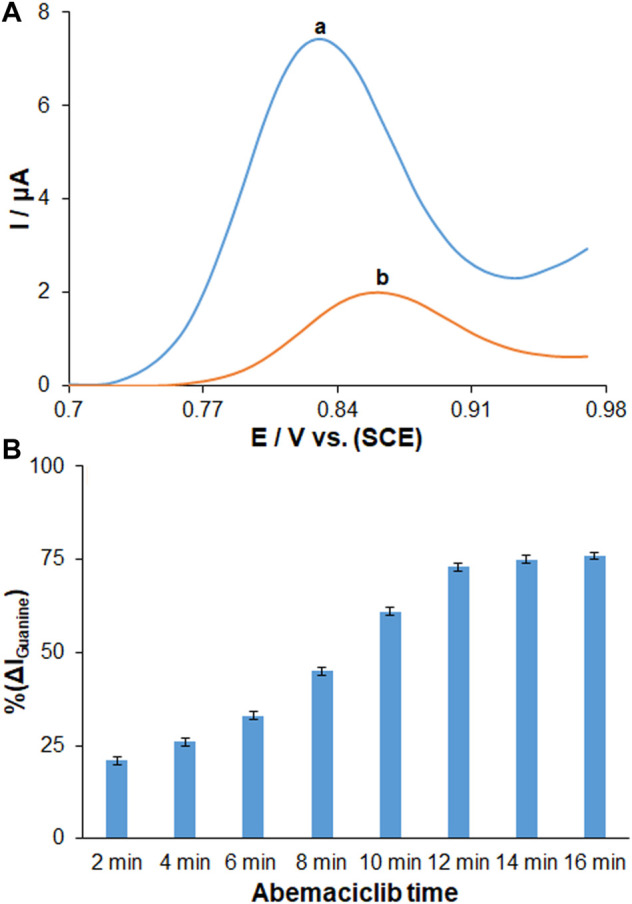
**(A)** DPVs in ABS (pH 4.8) for **(a)** ds-DNA/PP/Ce-doped H-NiO-ND/PGE and **(b)** ds-DNA/PP/Ce-doped H-NiO-ND/PGE after 30 min AMC interaction. **(B)** Histograms showing the effect of AMC interaction time on % change in magnitude of guanine signals measured for ds-DNA/PP/Ce-doped H-NiO-ND/PGE in ABS pH 4.8 after time durations ranging from 2 to 16 min (*n* = 3).

Given the improvement of biological interplay of matrix, other applications of biosensor have been studied to clarify the DNA-AMC interaction based on electrochemical technique considering intrinsic redox behavior of immobilized DNA. Figure 6A shows the DPVs in assessing the drug binding based on guanine oxidation signal in exposure to ds-DNA-immobilized PP/Ce-doped H-NiO-ND/PGE before and after AMC interaction (192.0 μg/ml). The voltammograms recorded for the guanine oxidation on the ds-DNA/PP/Ce-doped H-NiO-ND/PGE are shown in Figure 6Aa. The AMC-DNA binding significantly decreased the guanine signal, while the oxidation signal of drug was nearly disappeared (Figure 6Ab), which can be attributed to the ds-DNA density (Karimi-Maleha et al., 2022). Such reduced guanine signal was probably due to the electroactive guanine oxidizing group protection or guanine damage (Dogan-Topal and Ozkan, 2011b). Hence, the guanine may experience mutations due to AMC, leading to broken DNA strands particularly at guanine sites. To reach a more sensitive performance, the influence of incubation time of AMC was tested by exploring changes in the guanine oxidation ratio in ds-DNA/PP/Ce-doped H-NiO-ND/PGE at the AMC concentration of 220 μg/ml. Figure 6B shows a drop in the oxidation currents by the interaction times of 2–16 min, after which it becomes almost flat. Accordingly, the AMC-DNA interaction time of 12 min was selected for subsequent testing. The ds-DNA, PP and Ce-doped H-NiO-NDs were first used to modify the PEG. Then, the electrochemical signal of guanine was generated by the modified electrode for quantitative DNA detection. The electrochemical signal of guanine was finally decreased due to AMC, which was utilized for quantitative detection of AMC.

The effect of scan rate and heterogeneous rate constant of the electron transfer are usually investigated on sensors where the analytes are redoxed directly on the electrode surface, where the absorption or diffusion process is unclear. We have used here a biosensor that has been shown by other studies to be a surface absorption process. diffusion controlled process or adsorption controlled process was closely related to i plot with square root of scan rate or scan rate. Both processes (whether diffusion or adsorption) are scan rate dependent. In case of diffusion, it states that the number of particles diffuses through a cross-sectional unit area which is proportional to concentration differences across the area, while in case of the adsorption process electroactive species migrate from bulk phase to interface, Surface or pure adsorption controlled process is a phenomenon occurring due to the migration of molecules or particles from the bulk aqueous phase to the interface. On the contrary, in a diffusion controlled process, the molecules are transported from bulk phase to the subsurface (just below the interface). The molecules then move from subsurface to interface.

Therefore, a surface controlled process is characterized by the rate of molecule transport from the bulk to the interface, whereas a diffusion controlled process is explained by the rate of molecules diffuse initially into the subsurface and then finally onto the interface.

**FIGURE 7:**
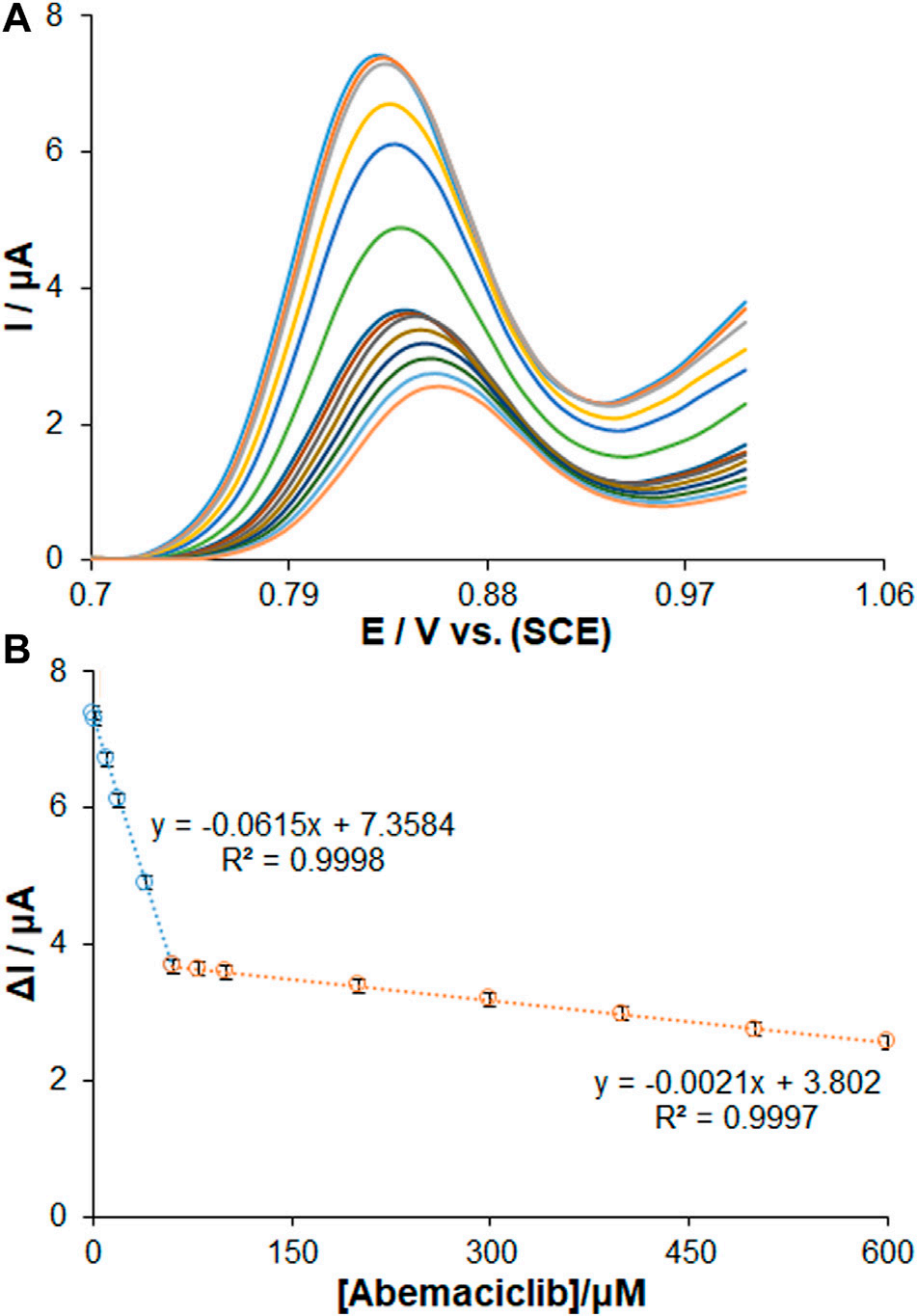
**(A)** DPVs for the interaction of AMC with ds-DNA/PP/Ce-doped H-NiO-ND/PGE. The oxidation signals of guanine after interaction with changing concentrations of AMC in the range of 0.01 µM–600.0 µM. Calibration plot for AMC in the concentration range of **(B)** 0.01–60.0 µM and 60.0–600.0 µM (*n* = 3).

The electrochemical behavior of ds-DNA/PP/Ce-doped H-NiO-ND/PGE was recognized for sensing AMC under optimized conditions. According to Figure 7A, the DPVs revealed a gradual decrease in the peak currents of guanine oxidation with increasing the AMC concentrations from 0.01 to 600.0 µM. Based on the standard curves, there was a linear AMC concentration range, in agreement with the count of active sites for lower and greater AMC concentrations, as seen in Figure 7B. The standard curves were recorded for different AMC concentrations of 0.01–60.0 µM and 60.0–600.0 µM (*n* = 3). The limit of detection (LOD) of AMC was determined to be 2.7 nM (3S_b_/m). This value of detection limit and the linear dynamic range for AMC observed for the ds-DNA/PP/Ce-doped H-NiO-ND/PGE are comparable and better than those obtained for several other previous studies (Table 1) (Margaryan et al., 2022; Turkovic et al., 2022; Martínez-Chávez et al., 2021). The detection limit of only several LC-MS/MS method developed for detection of AMC was superior to our sensor. When comparing with electrochemical methods, this method is expensive, sophisticated and multi-process techniques, with the need for sample preparation, pre-filtration and extraction as well as temperature monitoring.

**TABLE 1:** Comparison of major characteristics of various methods for the determination of AMC.

Methods	Dynamic ranges	Detection limits	Ref.
Liquid chromatography–mass spectrometry	0.2–500.0 nM	0.2 nM	Margaryan et al. (2022)
Liquid chromatography–mass spectrometry	15.0–3,000.0 ng ml^−1^	—	Turkovic et al. (2022)
Ultra performance liquid chromatography–mass spectrometry	1.0–600.0 ng ml^−1^	1.0 ng ml^−1^	(Martínez-Chávez, Tibben, deJong, Rosing, Schinkel, Beijnen)
Voltammetry	0.01–600.0 µM	2.7 nM	This work

The inset of standard curves shows the related regression equations. Five separate sensors measuring 10.0 µM of AMC (RSD = 1.73%) confirmed the excellent electrode reproducibility.

A lifetime study of the DNA biosensor was conducted to determine the stability of the DNA electrode. They were stored in the refrigerator at 4°C for a certain period throughout the studies. According to the result, The DPV for guanine oxidation was obtained to be 99.1% of its initial level for 10.0 µM of AMC after 3 months, thereby highlighting an impressive stability of as-fabricated biosensor. Even after 6 months of storage, 63.8% of its initial response could still be obtained. In addition, the ds-DNA/PP/Ce-doped H-NiO-ND/PGE exhibits interesting characteristics such as reusability of about 9 times and response time of 30 s when kept at 4°C.

**FIGURE 8:**
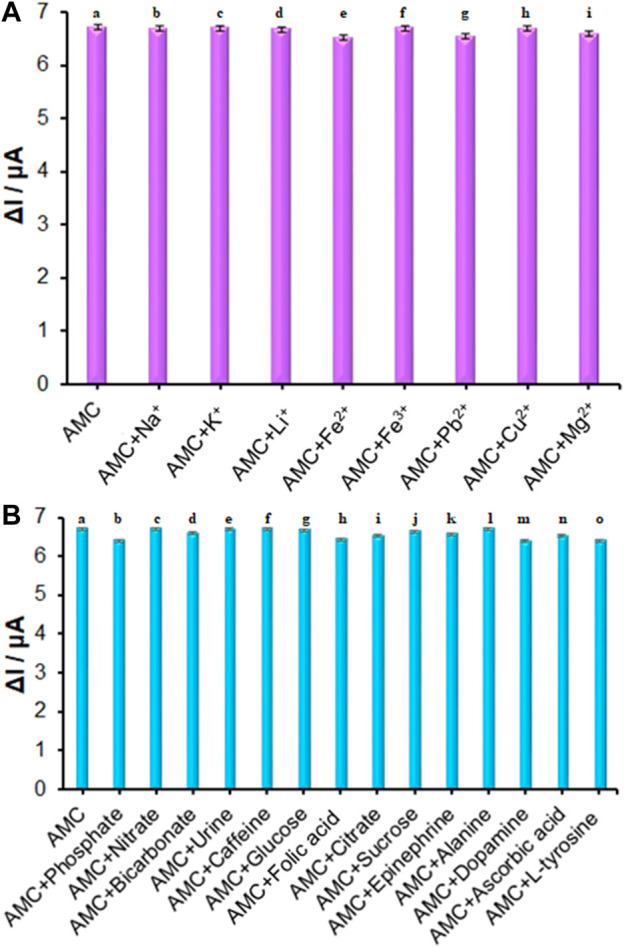
**(A)** The columns are the current change of ds-DNA/PP/Ce-doped H-NiO-ND/PGE in ABS (pH = 4.8) solution containing (a) 10.0 μM AMC, (b) a + 10.0 mM Na^+^, (c) a + 10.0 mM K^+^, (d) a + 10.0 mM Li^+^, (e) a + 10.0 mM Fe^2+^, (f) a + 10.0 mM Fe^3+^, (g) a + 10.0 mM Pb^2+^, (h) a + 10.0 mM Cu^2+^, (i) a + 10.0 mM Mg^2+^. **(B)** The columns are the current change of ds-DNA/PP/Ce-doped H-NiO-ND/PGE in ABS (pH = 4.8) solution containing (a) 10.0 μM AMC, (b) a + 10.0 mM phosphate, (c) a + 10.0 mM nitrate, (d) a + 10.0 mM bicarbonate, (e) a + 10.0 mM urine, (f) a + 10.0 mM caffeine, (g) a + 10.0 mM glucose, (h) a + 10.0 mM folic acid, (i) a + 10.0 mM citrate, (j) a + 10.0 mM sucrose, (k) a + 10.0 mM epinephrine, (l) a + 10.0 mM alanine, (m) a + 10.0 mM dopamine, (n) a + 10.0 mM ascorbic acid, and (o) a + 10.0 mM L-tyrosine.

The ds-DNA/PP/Ce-doped H-NiO-ND/PGE selectivity in sensing AMC was determined on the basis of DPVs recorded for modified electrode in the AMC solution of 10.0 μM in presence and absence of variable interferences, the data of which revealed that 1000-fold Na^+^, K^+^, Li^+^, Fe^2+^, Fe^3+^, Pb^2+^, Cu^2+^, Mg^2+^, Cl^−^, Br^−^ (Figure 8A), phosphate, nitrate, bicarbonate, urine, caffeine, glucose, folic acid, citrate, sucrose, epinephrine, alanine, dopamine, ascorbic acid and L-tyrosine (Figure 8B) had no significant interference (signal change<5%) in sensing AMC on ds-DNA/PP/Ce-doped H-NiO-ND/PGE surface. These changes may be illustrated by reasonable selectivity of ds-DNA/PP/Ce-doped H-NiO-ND/PGE for AMC.

### Potential pathway of ds-DNA-AMC interaction

The binding mechanism of ds-DNA-drug interaction can be explored by diverse techniques such as intercalation, nucleoside-analog incorporation, electrostatic interaction, DNA cleaving and groove binding (Hasanzadeh and Shadjou, 2016). The DPV, among these, is promising mean for the exploration of electrochemical profiles. The results determined the negative and positive shifts of the peak potentials for drug-DNA binding related to interactions of electrostatic and hydrophobic (intercalation), sequentially. Figure 7A shows the DPVs recorded for ds-DNA/PP/Ce-doped H-NiO-ND/PGE, the results of which revealed that a drop in peak current following AMC binding developed a shift in the guanine oxidation peak potential to further positive potentials with increasing the AMC concentrations, which relates to intercalation (Hatamluyi and Eshaghi, 2018).

### Real sample analysis

The potential of ds-DNA/PP/Ce-doped H-NiO-ND/PGE was tested in sensing AMC in injection, milk, serum and urine specimens based on a standard addition method. As seen in Table 2, the recovery rate was between 98.6% and 100.53% in detection of AMC using ds-DNA/PP/Ce-doped H-NiO-ND/PGE, highlighting outstanding performance of as-developed sensor. According to the result (Table 2), the t-calculated is lower than the t-critical, which means that the performance of the as-developed sensor is acceptable, and there is no systematic error.

**TABLE 2:** Determination of AMC in injection and human blood serum samples using ds-DNA/PP/Ce-doped H-NiO-ND/PGE (*n* = 5).

Sample	Detected (µM)	Added (µM)	Found (µM)^a^	RSD %	Recovery (%)	*t*-test
AMC injection	3.79	7.0	10.66 ± 0.12	1.13	98.79	2.41^c^
		17.0	20.82 ± 0.18	0.86	100.14	0.37^c^
Human blood serum	ND^b^	10.0	10.07 ± 0.14	1.39	100.7	1.11^c^
		20.0	20.09 ± 0.21	1.04	100.45	0.96^c^
Urine	ND^b^	5.0	4.93 ± 0.08	1.62	98.60	1.95^c^
		15.0	15.08 ± 0.13	0.86	100.53	1.37^c^
Milk	ND^b^	7.5	7.41 ± 0.11	1.48	98.80	1.82^c^
		12.5	12.39 ± 0.16	1.29	99.12	1.53^c^

a Mean ± standard deviation for *n* = 5.

b Not detect.

c *p* < 0.05, 95%.

### Intercalation docking

**FIGURE 9:**
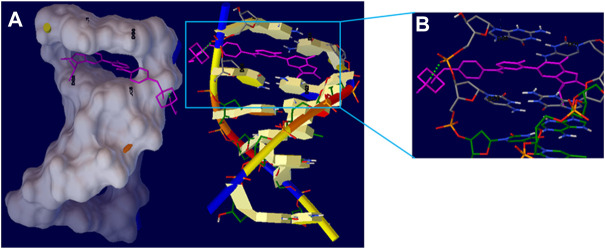
**(A)** The intercalation binding mode of Abemaciclib into DNA hexamer (1Z3F) (right) and surface view of Abemaciclib -hexamer docked complex (left). **(B)** The HB interaction of Abemaciclib to adjacent guanine residue 2 (DG2) and 6 (DG6) of DNA hexamer.

Figure 9A showed the docked AMC-hexamer configuration with the lowest binding energy (BE). As can be seen from Figure 9A, AMC intercalated into nitrogenous cytosine and guanine base pairs of the DNA receptor. The AMC-DNA complex is stabilized by π-π stacking interactions as well as intermolecular HBs with a BE of—10.96 kcal mol^−1^ and *K*_*i*_ 9.31 nM, according to the molecular docking research. It was discovered that the hydrogen atom in the AMC drug functioned as donor moieties in the formation of one O⋯H–N conventional hydrogen bond (HB) with the DNA base pairs [Hydrogen (H) 4 of AMC interacted with O 8 from guanine 6 (DG6) (Figure 9B)]. The AMC’s preferred intercalative binding was confirmed by the AMC’s greater negative BE with DNA hexamer.

## Conclusion

The present study developed a novel DNA-based electrochemical biosensor for determination of AMC, as an anticancer agent. The AMC intercalation into the ds-DNA guanine was applied as an analytical approach in the process of sensing. The ds-DNA/PP/Ce-doped H-NiO-ND/PGE had a significant sensing and catalytic performance towards variable AMC concentrations between 0.01 and 600.0 µM. Moreover, the ds-DNA/PP/Ce-doped H-NiO-ND/PGE was applicable selectively to detect AMC in the real specimens. Our findings may help to engineer and design a new highly sensitive electro-analytical instrument for the detection of anticancer agents.

## Data Availability

The original contributions presented in the study are included in the article/Supplementary Material, further inquiries can be directed to the corresponding authors.
