# Supplementary material for: Detection of abemaciclib, an anti-breast cancer agent, using a new electrochemical DNA biosensor
Source: Front Chem. 2022 Oct 21;10:980162. doi: 10.3389/fchem.2022.980162 (PMC9635563; doi:10.3389/fchem.2022.980162)
Supplement: Supplementary file 1 [file DataSheet1.docx]

**Detection of abemaciclib, an anti-breast cancer agent, using a new electrochemical DNA biosensor**

Zimeng Lei ^a,*^, Merim Alwan ^b^, Hassan Thoulfikar A. Alamir ^c^, Hussein Humedy Chlib Alkaaby ^d^, Sinan Subhi Farhan ^e^, Sura A. Awadh ^f^, Usama S. Altimari ^g^, Hawra'a Fadhel Abbas Al-Baghdady ^h^, Athmar Ali Kadhim ^i^, Maytham T. Qasim ^j^, Ali Hussein Adhab ^k^, Abuzar Nekuei ^l,*^

*^a^ School of International Education, Beijing University of Chemical Technology, 100029, Beijing, China*

*^b^ Medical Lab. Techniques department, College of Medical Technology, Al-Farahidi University, Iraq.*

*^c^ Faculty of pharmacy department of pharmaceutics, University of Al-Ameed, Iraq.*

*^d^ Al-Manara College For Medical Sciences, Maysan, Iraq.*

*^e^ The University of Mashreq, Baghdad, Iraq.*

*^f^ Department of anesthesia, Al-mustaqbal University, Babylon, Iraq.*

*^g^ Al-Nisour University College, Baghdad, Iraq.*

*^h^ College of Dentistry, the Islamic University, Najaf, Iraq.*

*^i^ Medical Laboratories Teachniques, Hilla University College Babylon, Iraq.*

*^j^ Department of Anesthesia, College of Health and Medical Technology, Al-Ayen University, Thi-Qar, Iraq.*

*^k^ Department of Medical Laboratory Technics, Al-Zahrawi University College, Karbala, Iraq.*

*^l^ Islamic Azad University of South Tehran Branch, Tehran, Iran.*

*Corresponding Authors: Emails:* [*2018100096@buct.edu.cn*](mailto:2018100096@buct.edu.cn)*, abuzaarnekuei@gmail.com*


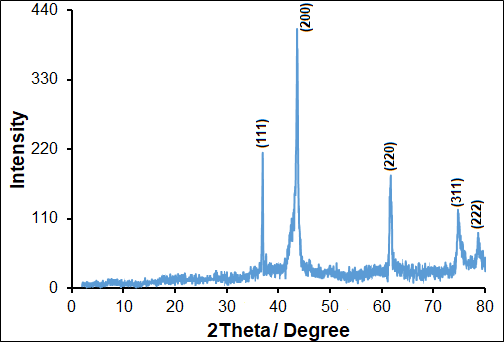


**Figure S1.** XRD pattern of Ce-doped H-NiO-ND.

**
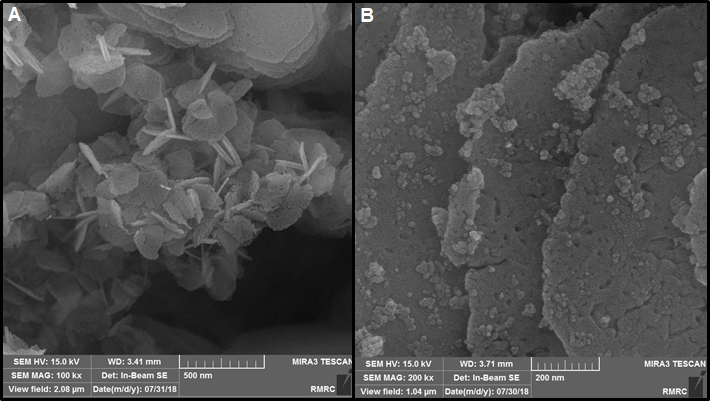
**

**Figure S2.** (A) FESEM image, (B) High resolution FESEM image of Ce-doped H-NiO-ND.

**
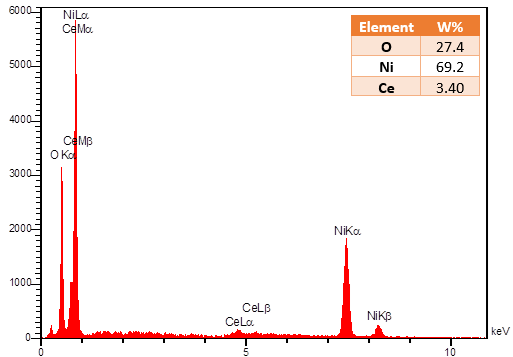
**

**Figure S3.** EDX spectra of Ce-doped H-NiO-ND.
